# Exposure to Per- and Polyfluoroalkyl Substances (PFASs) in Healthcare: Environmental and Clinical Insights

**DOI:** 10.3390/life15071057

**Published:** 2025-07-01

**Authors:** George Briassoulis, Stavroula Ilia, Efrossini Briassouli

**Affiliations:** 1 Postgraduate Program “Emergency and Intensive Care in Children, Adolescents and Young Adults”, School of Medicine, University of Crete, 71003 Heraklion, Greece; stavroula.ilia@uoc.gr; 2 Pediatric Intensive Care Unit, University Hospital, School of Medicine, University of Crete, 71110 Heraklion, Greece; 3 Second Department of Pediatrics, Aglaia Kiriakou Children’s Hospital, School of Medicine, National and Kapodistrian University of Athens, 11527 Athens, Greece; efroelesar@hotmail.com

**Keywords:** PFAS, per- and polyfluoroalkyl substances, environmental exposure, pediatric health, medical devices, endocrine disruption, immunotoxicity, bioaccumulation, regulatory policy, healthcare-associated exposure

## Abstract

Per- and polyfluoroalkyl substances (PFASs) are synthetic chemicals extensively used in various industries due to their unique physicochemical properties. Their persistence in the environment and potential for bioaccumulation have raised significant health concerns. This review aims to elucidate the sources, exposure pathways, toxicological effects, and regulatory measures related to PFASs, with a particular focus on pediatric populations and medical applications. A comprehensive narrative review was conducted using PubMed, Scopus, and Web of Science to identify peer-reviewed literature published between 2000 and 2025. The search focused on PFAS use in healthcare, environmental contamination, exposure pathways, health effects, and regulatory actions. Relevant studies, reports, and policy documents were screened and thematically synthesized by the authors to evaluate clinical and environmental risks, particularly in pediatric populations. PFAS exposure is linked to various adverse health effects, including immunotoxicity, endocrine disruption, metabolic disorders, and carcinogenicity. Children are particularly vulnerable due to developmental susceptibilities and exposure through medical devices and environmental sources. Regulatory measures are evolving, but gaps remain, especially concerning medical device applications. There is an urgent need for comprehensive strategies to monitor and mitigate PFAS exposure, particularly in vulnerable populations. Enhanced regulatory frameworks, safer alternatives in medical devices, and public health interventions are essential to address the challenges posed by PFASs.

## 1. Introduction

Per- and polyfluoroalkyl substances (PFASs) are a large class of anthropogenic chemicals that have been extensively manufactured and used globally since the 1950s. Their unique physicochemical properties—including resistance to heat, water, oil, and chemical degradation—have led to their incorporation into a wide range of industrial applications and consumer products, such as non-stick cookware, water-resistant textiles, food packaging, firefighting foams, and medical devices [1,2].

The same characteristics that make PFASs commercially valuable also contribute to their environmental and biological persistence. Owing to their strong carbon–fluorine bonds, PFASs degrade very slowly in natural systems and are therefore commonly referred to as “forever chemicals” [3]. These substances can accumulate in soil, water bodies, and living organisms, including humans, where they may exert adverse biological effects [4,5].

Emerging evidence has linked PFAS exposure to a range of health concerns, including immune dysfunction, metabolic disturbances, reproductive toxicity, and carcinogenicity [6,7,8]. Human exposure occurs primarily through contaminated food and water, inhalation of household dust, and dermal or parenteral contact with PFAS-containing products [9]. In healthcare settings, PFAS-coated devices such as catheters, guidewires, and implantable materials may represent a significant but under-recognized route of exposure, particularly among vulnerable populations such as neonates, children, and critically ill patients [10].

Despite growing awareness of the risks associated with PFASs, there are substantial gaps in monitoring, regulatory guidance, and effective remediation strategies. Analytical detection remains challenging due to the chemical diversity and low environmental concentrations of PFASs, and existing remediation methods often fall short in terms of cost-effectiveness and sustainability [11]. Furthermore, current regulatory frameworks are inconsistent and incomplete, particularly regarding cumulative exposure risks and long-term environmental impacts [12]. To safeguard public health, the European Union has recently taken regulatory action in the form of the revised Drinking Water Directive (EU 2020/2184), under which Member States must ensure PFAS concentrations in drinking water remain below 0.5 µg/L total PFAS and limit the sum of 20 priority PFAS to 0.1 µg/L by January 2026 [13,14,15].

In light of these challenges, comprehensive approaches are needed to assess PFASs distribution, evaluate health risks, and develop policies to reduce human exposure. This review provides a focused examination of PFAS exposure within clinical and environmental contexts, with particular attention paid to neonatal, pediatric, and intensive care settings where patients may face heightened vulnerability.

## 2. Materials and Methods

This narrative review was designed to synthesize current evidence on exposure to per- and polyfluoroalkyl substances (PFASs) through environmental contamination pathways relevant to child health, as well as potential exposure within healthcare settings—particularly in pediatric intensive care units (PICUs), neonatal intensive care units (NICUs), and general pediatric departments. The manuscript was jointly developed by three authors with expertise in pediatrics, pediatric intensive care, immunology, and environmental health.

### 2.1. Literature Search Strategy

A comprehensive literature search was conducted across PubMed, Scopus, and Web of Science for peer-reviewed publications dated between January 2000 and March 2025. Search terms combined Medical Subject Headings (MeSH) and keywords related to PFASs (“perfluoroalkyl substances”, “polyfluoroalkyl substances”, “PFOA”, “PFOS”, and “fluorochemicals”); healthcare exposure (“medical devices”, “hospital environment”, “patients”, “ICU”, “PICU”, “NICU”, and “iatrogenic exposure”); environmental contamination (“soil”, “d”, “residential exposure”, “biosolids”, and “industrial pollution”); and health outcomes (“pediatrics”, “immune dysfunction”, “liver disease”, “cancer”, “neurodevelopmental”, and “endocrine disruption”).

Only English language publications were included. In addition to peer-reviewed literature, gray literature was examined from relevant health agencies and regulatory bodies, including the European Chemicals Agency (ECHA), the U.S. Environmental Protection Agency (EPA), and the World Health Organization (WHO).

### 2.2. Study Selection

#### 2.2.1. Screening Process

**Figure 1:**
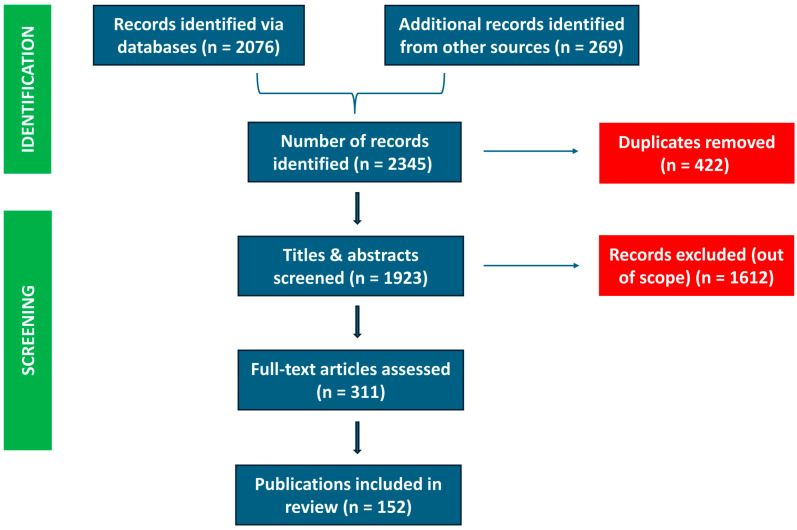
PRISMA-style flowchart summarizing the study selection process.

A PRISMA-style methodology was applied to guide the identification, screening, and inclusion of studies. A total of 2345 records were retrieved from initial searches across databases, health agencies, and regulatory sources. After removing 422 duplicates, 1923 records underwent title and abstract screening. Of these, 1612 were excluded for irrelevance or insufficient detail. The remaining 311 full-text articles were reviewed, resulting in 152 publications included in the final qualitative synthesis (Figure 1).

#### 2.2.2. Inclusion Criteria

Studies were eligible if they addressed one or more of the following topics:

(1) The presence or use of PFASs in clinical settings and medical devices;
(2) Environmental PFASs contamination in residential or community settings affecting pediatric populations;
(3) Health effects of PFASs exposure, particularly in children;
(4) Regulatory frameworks or mitigation strategies.

Included literature comprised systematic reviews, meta-analyses, observational and experimental studies, government reports, and policy papers. Case reports and opinion articles were excluded unless they contributed unique data or perspectives.

### 2.3. Data Extraction and Synthesis

Relevant data were extracted independently by the authors and categorized into the following thematic sections aligned with the review structure: (1) PFASs in medical applications, (2) environmental exposure and pediatric vulnerability, (3) toxicological health impacts, (4) mitigation strategies, and (5) regulatory measures. The findings were synthesized qualitatively, highlighting areas of scientific consensus, emerging evidence, and knowledge gaps. The review adheres to narrative synthesis guidelines appropriate for environmental health and toxicological risk assessment.

## 3. PFAS in Medical Applications

PFASs are extensively utilized in the medical technology sector due to their unique and highly desirable physicochemical properties. These include exceptional chemical and thermal stability, resistance to corrosion, low surface energy, biocompatibility, low friction (lubricity), and electrical insulation properties. Such characteristics make PFASs nearly indispensable in the manufacture, performance, and safety of various medical devices and diagnostic tools [1,2,12].

### 3.1. Essential Roles in Medical Devices

The functional demands of many medical applications often necessitate the use of PFAS-based materials. Fluoropolymers such as polytetrafluoroethylene (PTFE) and polyvinylidene fluoride (PVDF) are especially prominent in clinical settings due to their mechanical resilience, inertness, and sterility compatibility. These materials are used in both implantable and disposable medical devices. For example, PTFE coatings improve the biocompatibility of stents and orthopedic implants while minimizing biofilm formation and local tissue inflammation [16,17,18].

Applications include:

*Blood contact devices:* PFAS-based tubing and catheters are widely used for intravenous fluid delivery, blood transfusion, and vascular access procedures. Their inert surfaces reduce hemolysis and thrombogenicity [19].

*Implantable and surgical devices:* PTFE-based pledgets, sutures, and grafts offer enhanced durability and biocompatibility in cardiovascular, orthopedic, and reconstructive surgeries [20].

*Medical packaging and sterilization:* The high resistance of PFASs to chemical degradation and sterilization conditions makes them suitable for packaging sensitive instruments and maintaining sterile barriers [12,21].

*Wound care and barrier materials:* Surgical drapes, gowns, medical adhesives, and wound dressings often include fluorochemical coatings to enhance fluid repellency and reduce contamination risk [22].

In addition to general clinical use, fluoropolymer-based materials are indispensable in pediatric and neonatal care, especially in intensive care settings. For example, a randomized study in NICU neonates found that Teflon (PTFE) peripheral catheters performed comparably to other materials, supporting PTFE’s use for its chemical inertness and flexibility in fragile patients [23]. Fluoropolymers such as PTFE, FEP, and PVDF are widely employed in catheters, tubing, and filter membranes due to their low friction, biocompatibility, and sterilization resilience, which are critical attributes for life-supporting devices like neonatal catheters, feeding lines, endotracheal tubes, ECMO circuits, and dialysis systems [24]. Their excellent optical clarity further facilitates real-time monitoring in tiny conduits and diagnostic sensors—an essential feature in the NICU environment [25]. These stringent performance requirements underscore PFAS-based materials’ role in delivering safe and effective interventions for vulnerable pediatric patients.

### 3.2. PFAS in In Vitro Diagnostics and Analytical Instruments

PFASs are also critical to the functionality of in vitro diagnostic (IVD) reagents and instruments.

Surfactants in assay reagents help to stabilize proteins and enhance the detection of analytes such as magnesium or electrolytes in serum, plasma, and urine [26].

Heat transfer agents in diagnostic platforms help maintain temperature regulation, which is essential for biochemical reaction fidelity in clinical chemistry analyzers [27].

Instrument components made from fluoropolymers (e.g., PTFE/PVDF) are used in syringe pumps, tubing, valves, connectors, O-rings, and seals due to their high performance and chemical inertness [28,29].

### 3.3. Types and Classes of PFASs Used in Medical Technologies

A diverse range of perfluoroalkyl substances and polyfluoroalkyl substances are utilized in the medical sector, with applications spanning diagnostic reagents, medical devices, and specialty lubricants. The demand for chemical inertness, biocompatibility, mechanical durability, and resistance to extreme environmental conditions such as high temperatures, sterilization procedures, and reactive chemicals drives their inclusion [2,27].

(i) In Vitro Diagnostic (IVD) Reagents and Instruments

PFAS-based materials are integral to the structure and function of diagnostic equipment and reagent formulations. These compounds provide key functional properties—such as thermal conductivity, chemical resistance, and surfactant activity—that are crucial for ensuring accuracy and reliability in biochemical testing. Each substance plays a specific role within in vitro diagnostic (IVD) systems. Fluoroelastomers, valued for their flexibility and resistance to chemical degradation, are extensively used in gaskets, valves, seals, and other precision components of analytical instruments. Fluorinated surfactants and solvents are commonly incorporated into reagent formulations, employed as thermal control fluids in analyzers, and applied as surface treatments to reduce sample adhesion and prevent contamination [26,30]. While trifluoroacetic acid (TFA) meets the structural definition of a PFAS, its clinical and environmental relevance is likely minimal compared to other compounds in the healthcare context. Common PFASs used in IVD platforms are presented in Table 1.

(ii) Medical Devices

A wide spectrum of PFAS-containing materials is found in devices designed for direct patient contact. Fluoropolymers like polytetrafluoroethylene (PTFE) and polyvinylidene fluoride (PVDF) are widely utilized in tubing, reagent containers, pump elements, and fluid handling systems, primarily due to their strong resistance to chemicals and high thermal stability. Fluorinated ethylene propylene (FEP), a copolymer formed from hexafluoropropylene and tetrafluoroethylene, is commonly applied in medical tubing. Known for its durability, flexibility, biocompatibility, and excellent chemical resistance, FEP also offers high optical clarity. These characteristics make it especially well-suited for applications such as low-pressure microfluidic systems and catheter manufacturing. These materials contribute to the functionality, sterility, and durability of invasive and non-invasive devices. Common PFASs used in medical devices are presented in Table 2.

Hydrophobic fluoropolymer coatings and surface-bound fluorinated treatments are used to enhance device biocompatibility and reduce microbial adhesion. Many of these are proprietary blends with undisclosed compositions [10,12].

(iii) Specialty Fluorinated Lubricants and Suture Materials

Fluorinated lubricants are widely used in surgical instruments and precision mechanical components of medical devices, where minimal friction and high-temperature stability are required [31,32,33]. Additionally, PFAS-based suture materials are valued for their minimal tissue reactivity and enhanced knot security. Semi-fluorinated alkanes such as 1-(perfluorohexyl)octane and 1-(perfluorobutyl)-pentane are used in ophthalmic procedures and as biocompatible lubricants (Table 3).

### 3.4. Concerns About PFASs Leaching and Exposure

Despite their widespread utility, there is growing concern about the potential leaching of PFASs from medical devices into patients, particularly in neonatal, pediatric, and intensive care settings, where patients are often exposed to a variety of fluid infusions and multiple devices over extended durations. Studies have shown that trace amounts of PFASs can migrate from polymer coatings or plastic components into fluids administered intravenously or via enteral feeding, raising potential toxicological and bioaccumulation risks [5,34]. While the materials in ECMO systems are known to include PFASs, direct measurement of PFASs exposure in patients on ECMO has not yet been reported. More targeted research is needed to determine whether and how much exposure occurs through these devices.

Currently, alternatives to PFASs that match their performance and safety are limited. In many instances, proposed substitutes are short-chain PFAS derivatives—such as GenX—which, despite being increasingly used in medical applications, remain under-investigated and may carry similar health and environmental risks as legacy compounds. This underscores the urgent need for rigorous assessment of clinical PFAS exposures and the development of safer, non-fluorinated alternatives for medical use.

## 4. Environmental Contamination and Residential Exposure

While clinical settings represent a direct and often underappreciated route of PFASs exposure, widespread environmental contamination also contributes substantially to the human PFASs burden, particularly among children. PFASs are highly persistent in natural environments due to their chemical stability and resistance to degradation. Once released into the environment—typically via industrial emissions, wastewater discharge, or landfill leachates—these compounds can accumulate in soil, surface and groundwater, and biota [2,3,9].

### 4.1. Environmental Sources and Pathways of Exposure

**Figure 2:**
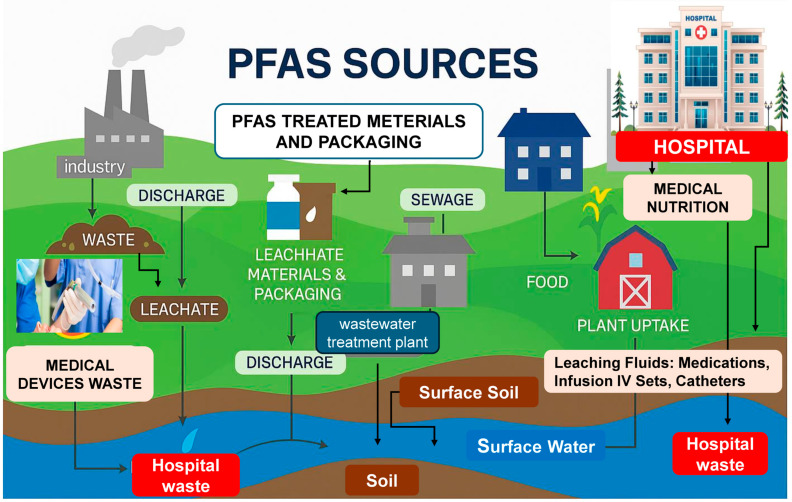
Environmental sources and pathways of exposure to PFASs.

PFASs are now ubiquitously found across multiple environmental media, with emerging data highlighting their persistence and reach (Figure 2). A comprehensive global survey published in 2024 analyzed over 45,000 surface and groundwater samples and found that approximately 31% of samples exceeded PFAS thresholds deemed harmful to human health [35]. In North America, PFASs have been detected in 45% of tap water sources surveyed by the U.S. Geological Survey [2], while a 2024 survey from the U.S. Environmental Protection Agency indicated that 70 million Americans receive drinking water containing detectable PFAS levels [3].

Recent European studies reinforce this prevalence: monitoring data reported in late 2024 showed PFOS concentrations above regulatory limits in a significant proportion of surface water sites across the EU [36]. Additionally, nearly 69% of groundwater samples in regions with stringent regulations, such as Canada, surpassed national PFAS thresholds [35].

In agricultural zones, biosolid-amended soils have been reported to contain PFAS levels as high as 300 µg/kg, which can result in crop uptake and subsequent human exposure [37]. Airborne pathways also contribute to far-reaching contamination: PFAS precursors in atmospheric transport deposit via rain and dust even in remote regions [38].

Indoor environments are similarly affected, particularly spaces frequented by children. Household dust in homes with PFAS-treated consumer items (e.g., textiles, waterproof clothing) has been identified as a chronic source of exposure via inhalation and ingestion [39]. These findings underscore the multifaceted and persistent nature of PFAS contamination across global media, emphasizing the need for targeted interventions and monitoring efforts.

*Surface and groundwater:* Industrial sites producing or using PFASs (e.g., fluorochemical manufacturing, metal plating, textile treatment, and firefighting training facilities) are primary point sources. Wastewater treatment plants, unable to effectively remove PFASs, also contribute to surface water contamination. Their high solubility and mobility allow PFASs to travel far from the original source and enter drinking water supplies [40,41].

*Soil and agricultural contamination:* Application of biosolids or contaminated irrigation water introduces PFASs into soil systems, from which they can leach into crops or groundwater [37,42].

*Air and atmospheric transport:* Volatile PFAS precursors can become airborne and deposit through precipitation or dust, resulting in widespread distribution even in remote areas [43,44].

*Indoor environments:* Dust from PFAS-treated consumer goods (e.g., carpets, upholstery, waterproof clothing) contributes to chronic low-level inhalational or ingestion exposure, particularly in children [45].

### 4.2. PFASs in European Residential Areas

Recent mapping efforts have revealed over 17,000 PFASs contaminated sites across Europe, with high-density clusters in industrial regions of Belgium, Germany, the Netherlands, and Italy. Residential communities near fluorochemical plants or military installations—where aqueous film-forming foams (AFFFs) have been used—face especially elevated risks. National responses vary widely, with some countries enacting strict drinking water limits and others lacking systematic monitoring [46].

Recognizing critical exposure pathways for children and infants, we have summarized these in Table 4. Environmental sources, specifically contaminated drinking water and household dust, consistently present the highest and most well-documented exposure risks [47]. Breastfeeding and prenatal transfer are significant sources of PFAS exposure, particularly during infancy and early childhood when physiological vulnerability and cumulative exposure risks are highest. In contrast, exposure via medical devices, such as PTFE tubing, catheters, and ECMO circuits, remains largely unquantified. Although these clinical materials inherently contain PFASs due to their chemical benefits, there is a notable lack of empirical data assessing direct patient exposure from such sources. Indeed, neonatal dried blood spot (DBS) studies confirm PFASs presence immediately after birth, indicating effective placental transfer [48,49], and further research has demonstrated detectable PFAS concentrations in neonatal plasma [50,51]. Additionally, preliminary clinical biomonitoring suggests that hospital-administered procedures, including IV infusions, catheter use, and respiratory support in neonatal and pediatric intensive care, may elevate PFAS levels via administered fluids [52]. However, quantitative assessments of PFAS leaching from ICU tubing and other in-hospital devices are still absent, emphasizing the urgent need for rigorous in-hospital exposure studies.

### 4.3. Vulnerability in Pediatric Populations

Children are particularly vulnerable to PFAS exposure due to their developing organs, higher metabolic rates, greater intake of air, food, and water per unit body weight, and behaviors such as hand-to-mouth contact that increase exposure risk. Biomonitoring data show that young children frequently have higher serum PFAS concentrations than adults, particularly in environmentally contaminated areas [5,42,43,60].

A recent analysis from the CHARGE study (2009–2017) reported median PFOS serum levels of 8.1 ng/mL (range 5.0–31.5) in children aged 2–5—significantly exceeding levels found in their mothers. These elevated levels likely result from exposure through breastfeeding, dust ingestion, and age-specific behaviors [61]. Norwegian cohort data (2023) confirmed that children under 12 had notably higher concentrations of PFOS, PFOA, PFHxS, and PFHpS than older children, with about 20% exceeding current safety thresholds [62]. A 2023 physiologically based pharmacokinetic (PBPK) model estimated mean PFOS levels of 7–12 ng/mL and PFOA levels of 3–6 ng/mL in children aged 6–12, aligning with biomonitoring observations [63].

Infants and toddlers face heightened exposure risks, particularly via breastfeeding. Studies show that PFOA and PFOS are present in human milk, resulting in the highest daily PFAS intake during exclusive breastfeeding, with strong correlations to infant plasma concentrations [52]. The duration and exclusivity of breastfeeding are key determinants; monthly PFAS serum increases reach up to 30% with exclusive breastfeeding and have lower rates with partial breastfeeding, whilst levels drop after weaning [64]. A 2023 study observed that toddlers had higher total PFAS concentrations than their mothers, with each additional month of breastfeeding associated with plasma PFOS, PFOA, and PFHpS increases of 3.3%, 4.7%, and 6.1%, respectively [65]. These early-life exposures raise concern given established links between prenatal and postnatal PFASs exposure and adverse health outcomes, including low birth weight, fetal growth restriction, thyroid and kidney dysfunction, and neurodevelopmental issues such as ADHD and reduced cognitive performance [66]. Prenatal PFAS-associated DNA methylation changes identified in the Project Viva cohort—specifically, 435 CpG sites altered in cord blood and persisting into childhood—include loci associated with neural, metabolic, and cognitive health [67]. Such epigenetic modifications may influence gene expression pathways critical to neurodevelopment, suggesting a potential mechanistic link between these DNA alterations and observed neurobehavioral risks.

Although comparative data from clinical settings are still lacking, public health agencies—including the CDC and ATSDR—have emphasized the need to support healthcare professionals with educational resources. These materials should help clinicians understand PFAS exposure pathways, potential health effects, and the limitations of testing and follow-up. Pediatric care in particular would benefit from this knowledge as early interventions can reduce cumulative exposure in vulnerable populations [68].

### 4.4. Importance of Surveillance and Remediation

Current remediation techniques such as activated carbon filtration, high-pressure membranes, and ion exchange resins are partially effective for long-chain PFASs but may fail for short-chain variants. Emerging technologies like electrochemical oxidation, supercritical water treatment, and bioremediation are promising but not yet widely available [69].

Surveillance systems for PFASs in soil, water, and biota should be harmonized across Europe and globally to track contamination patterns and inform mitigation strategies. Pediatric health institutions, especially those serving at-risk populations, may benefit from incorporating environmental history assessments and regional PFASs data into their patient intake procedures [70].

## 5. Toxicological Effects of PFAS Exposure

Per- and polyfluoroalkyl substances are linked to a broad spectrum of adverse health effects due to their ability to bioaccumulate and interact with lipid, protein, and hormone pathways. These effects may manifest at both low and high levels of exposure and are of particular concern in vulnerable populations such as children, pregnant women, and chronically ill individuals

### 5.1. Hematological and Cardiovascular Effects

PFASs impact platelet function by altering plasma membrane dynamics and calcium signaling, leading to enhanced adhesion, aggregation, and thrombus formation—thus elevating pro-thrombotic risk, especially in individuals with pre-existing vascular conditions [71,72]. Mechanistic studies reveal that PFAS engagement with platelet GPIbα triggers calcium influx, Akt activation, and αIIbβ3 integrin signaling, culminating in procoagulant platelet formation. Animal models exposed to PFASs show increased thrombus development and ischemic stroke, suggesting potential targets (e.g., GPIbα) for mitigating PFAS-induced thrombosis [73].

Epidemiological analyses demonstrate dose–response relationships between mixed PFAS exposures—and PFOA in particular—and alterations in hematologic indices, including hematocrit, hemoglobin, platelets, WBC count, and inflammatory markers, implicating pathways like JAK-STAT, apoptosis, Th17 differentiation, and atherosclerosis [74]. PFASs have also been associated with metabolic and cardiac effects—such as elevated cholesterol/triglycerides, altered electrophysiology, and increased coronary events [75]. Notably, higher serum PFHxS and PFDeA concentrations have been linked to increased abdominal aortic calcification (AAC) risk, with a dose-dependent relationship observed for PFHxS; furthermore, age and PFHxS levels were the strongest predictors in SHAP-XGBoost models [76].

### 5.2. Liver and Metabolic Disruption

The liver is a major target of PFAS toxicity. Human and animal studies consistently show elevated liver enzymes, hepatic fat accumulation, and disruptions in lipid and bile acid metabolism, even among children without traditional metabolic risk factors [77]. These effects contribute to the development of metabolic-associated fatty liver disease (MAFLD) and non-alcoholic fatty liver disease (NAFLD). Metabolomics analyses further reveal PFAS-induced perturbations in glucose and lipid metabolic pathways [78].

Legacy PFAS mixtures have been shown to primarily disrupt amino acid and lipid metabolism, while newer PFAS compounds exert broader effects across metabolic pathways, including those involved in carbohydrate processing, vitamin/cofactor utilization, and endocrine regulation [79]. In the Boston Birth Cohort, a metabolome-wide analysis of 590 mother–infant pairs identified PFAS-associated changes in 331 metabolites and 18 pathways, most notably involving amino acids and lipids linked to fetal growth restriction, cardiovascular risk, and insulin resistance. Carnitine pathway disruptions, associated with mitochondrial β-oxidation, and altered neurotransmitter metabolites were also implicated, with stronger effects observed in males [80].

Early-life PFAS exposure may also predispose children to obesity and overweight (OWO). Higher exposure to both individual PFAS compounds and their mixtures was associated with increased risk of OWO, especially in older children and in those whose mothers did not have pre-pregnancy OWO. Synergistic effects between PFAS exposure and maternal OWO were particularly evident during adolescence [81]. Additionally, a twofold increase in PFAS levels at 6 and 12 months was associated with a 10–15% reduction in resistin levels by age 9, suggesting long-term metabolic impact [82]. Experimental data also link PFOSs to impaired maternal–fetal metabolism and fetal hematopoiesis, raising concerns about immunometabolic consequences of environmental PFASs exposure during gestation [83].

### 5.3. Immune System Impairment

Emerging evidence confirms that PFASs are immunotoxic, particularly in children. Exposure has been linked to diminished antibody responses to routine vaccinations and an increased susceptibility to respiratory infections, driven by the dysregulation of cytokine profiles and systemic effects on both innate and adaptive immunity [6,84]. Children with pre-existing immunodeficiencies or chronic illnesses may be especially prone to these impacts [85].

A recent meta-analysis reinforces these concerns, finding possible associations—particularly with PFOS, PFOA, PFHxS, and PFNA—between PFAS exposure and higher infection risk in infants and children, although the evidence for suppressed antibody responses remains modest [86]. Supporting this, the Hokkaido Study (2689 mother–child pairs) reported that prenatal PFAS exposure was linked to increased allergy and infectious disease prevalence up to age 7, consistent with immunosuppressive effects observed at ages 2 and 4 [87].

### 5.4. Endocrine Disruption

PFAS are well-documented endocrine disruptors that interfere with critical hormonal pathways—including thyroid, adrenal, and reproductive systems. Exposure during sensitive developmental periods has been associated with altered puberty timing, impaired fertility, and adverse pregnancy outcomes [88]. Additionally, disruptions in hormonal signaling may contribute to increased risks for endocrine-related cancers.

Evidence from a study near Antwerp, Belgium, where a 3M facility has released PFASs, mainly PFOS, since 1971, highlights real-world impacts. Among adolescents in the area, exposure to PFHxS and PFOA was inversely associated with LH and testosterone levels in males, while sex hormone binding globulin (SHBG) was positively linked with PFDA, PFNA, PFHxS, and PFOS. In both sexes, PFOS and PFOA were negatively associated with growth and pubertal milestones, including height, growth spurts, and breast development. FT3 levels in females showed a negative association with branched PFOS isomers, indicating sex-specific endocrine disruption across multiple hormonal axes [89].

Per- and polyfluoroalkyl substances have been found to suppress the expression of critical genes involved in steroid hormone biosynthesis, such as the steroidogenic acute regulatory protein, several cytochrome P450 enzymes (CYP11A1, CYP17A1), and 3β- and 17β-hydroxysteroid dehydrogenases. Furthermore, PFASs can directly damage developing germ cells—including spermatogonia and oocytes—thereby potentially compromising reproductive capacity [90].

### 5.5. Neurodevelopmental and Mental Health Effects

Evidence is accumulating that PFAS exposure may impact neurodevelopmental trajectories and mental health. Associations have been reported with attention deficit hyperactivity disorder (ADHD), lower cognitive scores, and increased risk of depression, possibly mediated through oxidative stress and neurotransmitter pathway disruption [78,91].

Early-life PFAS exposure poses significant neurodevelopmental risks, given the vulnerability of the developing brain. A comprehensive review of 61 studies (2008–March 2024), including 35 from the past five years, consistently linked prenatal and early childhood PFAS exposure to poorer cognitive, motor, and language outcomes in infants, as well as increased behavioral issues in childhood [92]. The Norwegian Mother, Father and Child Cohort Study reported that prenatal PFOA exposure was associated with greater risks of ADHD and ASD among 821 ADHD cases, 400 autism spectrum disorder (ASD) cases, and 980 controls; inverse associations for some PFASs and mixtures likely reflect residual confounding rather than protective effects [93]. Additionally, elevated prenatal PFOS, PFOA, and PFHxS levels were linked to 1.6–2.0 point reductions in boys’ performance IQ per log-unit increase; furthermore, PFAS exposure was also associated with a decline in executive function, although not clearly with behavioral difficulties [94].

Cohort studies reinforce these findings across different settings and compounds. In Atlanta, combined PFAS and PBDE exposures were linked to increased internalizing and externalizing behaviors among 1–5-year-olds [95], and novel PFASs such as GenX have been implicated in neurodevelopmental disruption [96]. The MIREC study found that higher prenatal PFOA, PFOS, and PFHxS levels were associated specifically with lower performance IQ in boys, based on WPPSI-III and BRIEF-P assessments [97]. Similarly, the Sheyang Mini Birth Cohort Study (*n* = 327, age 7) reported that elevated prenatal PFAS levels corresponded with reduced full-scale IQ, independent of fetal thyroid function [98]. Collectively, this evidence underscores domain-specific and sex-specific vulnerabilities in early neurodevelopment associated with PFAS exposure.

### 5.6. Carcinogenicity

The IARC Monograph 135 (2023) classifies PFOA as a Group 1 carcinogen (carcinogenic to humans) and PFOS as Group 2B (possibly carcinogenic) [99,100]. PFOA has been linked to testicular and kidney cancers in epidemiologic studies, supported by strong mechanistic and animal evidence [100]. PFOS remains possible, based on mechanistic data but with limited evidence in people. Proposed carcinogenic mechanisms include activation of peroxisome proliferator-activated receptor alpha (PPAR-α), which influences lipid metabolism, cell proliferation, and inflammation—pathways that may contribute to tumor development, B genotoxic effects, endocrine disruption, and epigenetic alterations [101].

Environmental and clinical investigations into PFAS exposure have revealed emerging links to childhood cancers and tumor-associated molecular disruptions [102]. In California, maternal serum PFOS and PFOA levels (range: 5–22.9 ng/mL and 2–6.7 ng/mL, respectively) were suggestively linked with nonastrocytoma gliomas, acute myeloid leukemia, Wilms tumors, and embryonal cancers, particularly among children born to mothers from Mexico (AOR 1.07–1.26) [103]. Among U.S.-born mothers’ children, PFOS levels were tied to a 15% increased odds of unilateral retinoblastoma per interquartile increase, rising to 71% among those above the mean; PFOA showed similar trends (aOR 1.06–1.41) [50]. Further, household dust PFAS exposure, including N-ethyl perfluorooctane sulfonamido acetic acid (EtFOSAA), was associated with increased childhood acute lymphoblastic leukemia risk [104]. Prenatal exposure to N-methyl-perfluorooctane sulfonamidoacetic acid in the Finnish Maternity Cohort doubled the odds of acute lymphoblastic leukemia in offspring (OR log_2_ = 1.22; high-level OR = 2.52) [105].

At the molecular level, PFASs have been shown to disrupt key metabolic and epigenetic pathways. Late-pregnancy PFAS exposure, alongside DDT, altered amino acid metabolism—impacting lysine, tryptophan, histidine, and branched-chain pathways—potentially undermining immune function and promoting oncogenesis [106]. In vitro and bioinformatic studies identified PFAS interactions with core cancer pathways, including cell cycle regulation, inflammation, metabolism, and V repair, highlighting targets such as CDC20, MYC, BIRC5, PTEN, and IL6 [107]. PFASs were also linked to dysregulation in core genes for hepatocellular (APOA1 and ESR1) [108] and breast cancer (PPARG, CD36, and FABP4) [109] via PPI network and transcriptomic analyses. Biomarkers like altered miR-148b-3p and miR-29a-3p expression (Teen-LABs and Rhea cohorts) have been correlated with PFAS exposure, suggesting connections to cancer and chronic inflammation [110], while prenatal exposure was associated with DNA methylation changes in genes related to cancer, neurological, cardiovascular, and renal health (Project Viva) [67]. Remarkably, various PFASs—including short-chain compounds—have been detected in human glioma samples, constituting 52% of total PFASs in tumor tissue and underscoring blood–brain barrier permeability [111]. 

### 5.7. Multi-Organ Metabolic Disruption

Comprehensive metabolomic investigations reveal that PFASs exposure alters key metabolic pathways, including amino acid, nucleotide, carbohydrate, and vitamin metabolism [112], disrupting mitochondrial function and cellular energy homeostasis, particularly among children and adolescents with chronic exposure [113]. This systemic disruption has potential implications for multi-drug resistance (MDR): notably, 6:2 Cl-PFESA, a PFOS substitute, has been shown to influence MDR mechanisms, underlining the need for rigorous evaluations of its carcinogenic potential [114].

PFASs have also been implicated in neurochemical and endocrine disruption. Utilizing an adverse outcome pathway (AOP) framework, a review of 271 studies identified reactive oxygen species (ROS) generation as the primary molecular initiating event, triggering oxidative stress, neuroinflammation, apoptosis, dysregulation of Ca^2+^ signaling, neurotransmitters (glutamate, dopamine, and serotonin), synaptic dysfunction, and ultimately cognitive and neurodevelopmental impairments including ASD and ADHD [115]. Additionally, PFAS exposure has been linked to thyroid hormone disruption [116], with Na+/I- symporter (NIS) inhibition identified as a secondary initiating event that leads to downstream effects on hormone synthesis, transport, and receptor binding, mechanisms culminating in myelination deficits and neurological dysfunction [115]. These molecular pathways—marked by mitochondrial impairment, excitotoxicity via NMDA-R overactivation, and inflammatory cascades—provide mechanistic insight into the potential neurodegenerative and developmental risks posed by PFASs, reinforcing the scientific basis for regulatory and public health action [117].

### 5.8. Early-Life and Dietary Exposure

**Figure 3:**
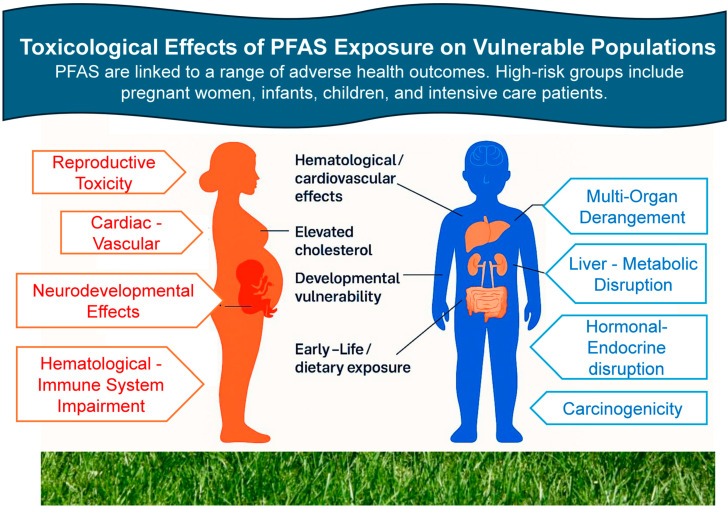
Toxicological effects of PFASs exposure are associated with various adverse health outcomes. Vulnerable populations include children, pregnant women, and chronically ill subjects.

Children and infants are particularly vulnerable due to physiological immaturity and dietary habits (Figure 3). PFASs and phthalates have been detected in infant formula, baby bottles, and teats [22]. These early exposures may contribute to endocrine and developmental disorders. Dietary studies show cumulative expo, water, and packaging exposure in pregnant women and young adults [118].

Early-life exposure to PFASs—including in utero, through breastfeeding, and via early diet—is increasingly recognized for its potential impact on child development. A PBPK model applied to the HELIX cohort (1239 mother–child pairs) estimated that PFAS levels peak around age 2, with initial predominance of PFOA over PFOS before reversing later in childhood. Average concentrations declined by age 8 (PFOA: 3.1 → 1.9 ng/mL; PFOS: 4.8 → 3.6 ng/mL), with exposure driven by prenatal transfer, breastfeeding (up to 2–5 years), and food ingestion [63]. Breastmilk studies show PFOA, PFOS, PFHxS, PFNA, PFDA, and PFUnDA present in over 90% of maternal samples, with PFOS transfer efficiencies of ~7% at 9.5 months and ~6% at 11.5 months [119]. Exclusive breastfeeding correlates with up to a 30% monthly increase in infant PFAS levels, while toddlers show higher PFASs than mothers—with plasma PFOS, PFOA, and PFHpS increasing by 3.3%, 4.7%, and 6.1% per month of breastfeeding, respectively [52,64,65,81,119].

These chemical exposures have tangible health impacts as the PFAS burden at 6–12 months correlates with a lower weight-for-length z-score (β = −0.20; 95% CI −0.35, −0.04), especially in females [120], and in utero PFAS exposure associates with reduced birth anthropometrics—birth weight decreased by up to 181 g per ng/mL increase in PFHpS and 24 g per ln-unit of PFDA, with additional effects on length, head circumference, gestational age, preterm birth, and small-for-gestational-age outcomes [121,122]. Collectively, these findings underline how prenatal and early-postnatal PFAS exposures can influence growth and health trajectories from the earliest stages of life.

## 6. Strategies for Monitoring and Mitigation of PFAS Exposure in Pediatric Patients

A strategic response to PFAS exposure in pediatric care must encompass prevention, surveillance, and policy-driven mitigation across clinical and environmental contexts.

### 6.1. Medical Device Review

Institutions should assess PFASs content in medical products, prioritize PFASs-free alternatives when available, and collaborate with manufacturers to develop safer materials. Priority should be given to high-use items such as infusion lines, catheters, and feeding tubes [71].

### 6.2. Environmental Monitoring and Patient Screening

Routine monitoring of water and soil in communities near industrial sites can guide interventions [5,123]. For patients admitted to PICUs, biomonitoring at admission and discharge could provide insight into iatrogenic exposure. While not yet standard practice, targeted PFASs testing may be warranted in high-risk scenarios [124].

### 6.3. Policy Implementation and Circular Economy Integration

**Figure 4:**
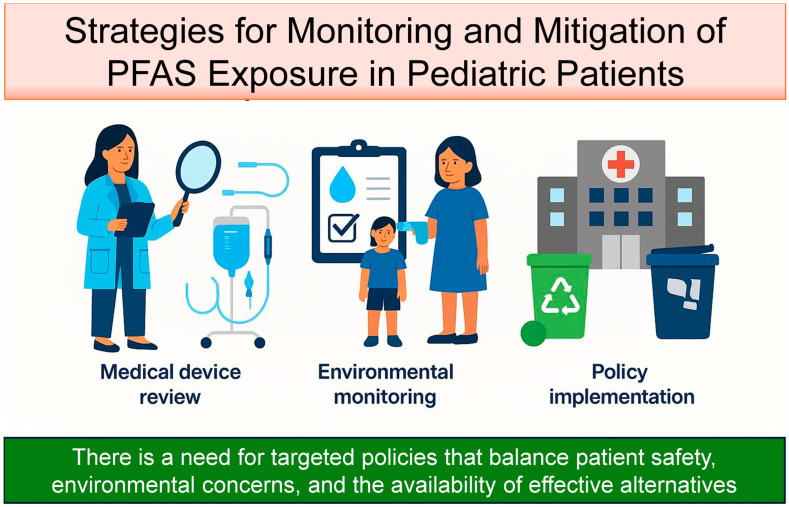
Strategies for monitoring and mitigation of PFAS exposure in pediatric patients.

Hospitals should adopt procurement policies that favor low-PFASs or PFASs-free equipment. When devices are confirmed free of PFASs, circular economy principles—such as sterilization and reuse—can be implemented to reduce environmental waste [125]. Appropriate waste management protocols must be in place for PFASs-containing items (Figure 4).

## 7. Regulatory Measures and Guidelines

### 7.1. European Union Initiatives

The European Union (EU) has been proactive in addressing the risks associated with PFASs. In January 2023, a comprehensive proposal was submitted by Germany, the Netherlands, Denmark, Sweden, and Norway to the European Chemicals Agency (ECHA) under the Registration, Evaluation, Authorisation, and Restriction of Chemicals (REACH) regulation [126]. This proposal aims to restrict the manufacture, market placement, and use of PFASs, with certain time-limited derogations for specific applications where alternatives are not yet available. The restriction is anticipated to come into force no earlier than 2029–2030, allowing industries time to transition to safer alternatives.

In parallel, the revised Drinking Water Directive (EU 2020/2184) mandates enforceable limits for PFASs in drinking water—0.1 µg/L (100 ng/L) for the sum of 20 PFAS compounds and 0.5 µg/L (500 ng/L) for total PFASs—with compliance required by January 12, 2026 [13]. Technical guidelines for monitoring have also been issued to support implementation [127].

### 7.2. United States Environmental Protection Agency (EPA) Actions

In the United States, the Environmental Protection Agency (EPA) has taken significant steps to regulate PFASs. In April 2024, the EPA designated PFOA and PFOS, including their salts and structural isomers, as hazardous substances under the Comprehensive Environmental Response, Compensation, and Liability Act (CERCLA) [128]. Additionally, the EPA announced enforceable maximum contaminant levels (MCLs) for six PFAS compounds in drinking water, setting limits at four parts-per-trillion (ppt) for PFOA and PFOS, and establishing a cumulative hazard index of 1.0 for PFNA, PFHxS, PFBS, and GenX chemicals [129].

### 7.3. Gaps in Medical Device Regulation

Despite these regulatory advancements, specific regulations addressing PFASs in medical devices remain limited. The medical technology sector relies on PFASs for their unique properties, including chemical resistance, heat resistance, durability, and biocompatibility. Given the essential role of PFASs in certain medical applications, there is a need for targeted policies that balance patient safety, environmental concerns, and the availability of effective alternatives [71].

Despite strong legislative intent, implementing REACH restrictions in medical and diagnostic contexts poses significant challenges. The supply chain for PFAS-based materials, such as PTFE in tubing and catheters, is tightly integrated into medical device design. Evidence suggests that even if exempted, medical device manufacturers must reassess conformity under Medical Device Regulation (MDR)/Medical Device Directive (MDD), facing potential shortages and the need for costly redesigns after REACH changes [130]. Monitoring capacity and regulatory auditing for PFASs use in medical devices remains unclear. We recommend targeted guidance on substitution timelines, assistance for conformity reassessment, and inclusion of PFASs in the EU’s MDR framework.

While the EPA’s focus on environmental exposure is commendable, current US regulations do not address PFASs within medical devices, which may inadvertently serve as a significant exposure source in clinical settings [131]. Moreover, state-level regulations (e.g., California, Michigan) impose additional complexity for medical facilities operating across jurisdictions. Federally, PFASs regulation under the Toxic Substances Control Act (TSCA) may allow for the fast-tracking of manufacturing exemptions (semiconductor industry loopholes) [69]. 

### 7.4. Suggestions for Pediatric Care Environments

*Risk-based material substitution:* Hospitals and pediatric care units should proactively conduct PFAS gap analyses in medical devices, collaborating with suppliers to identify safer, performance-equivalent alternatives [132].

*Regulatory monitoring and compliance:* Hospital risk management and procurement teams should incorporate PFAS regulatory tracking as part of their quality systems (under MDR or the US FDA’s artificial intelligence/machine learning guidance) [133].

*Cross-sector engagement:* Professional bodies (European Federation of Clinical Chemistry and Laboratory Medicine) should partner with regulators and manufacturers to develop transition roadmaps for PFAS-free medical devices in neonatal and pediatric care, focusing first on high-exposure items such as ECMO circuits and IV tubing [134].

*Transparency policies:* Pediatric hospitals could mandate PFAS content disclosure in purchasing contracts, driving accountability and incentivizing innovation [135]. 

*Degradation strategies*: Ongoing advances in biotechnology present a viable and sustainable approach to PFAS remediation, with the potential to mitigate both environmental impacts and associated public health risks [136].

### 7.5. Advancing a Circular Economy in Pediatric Healthcare

A circular economy model in pediatric care can substantially minimize PFAS-related environmental contamination and reduce exposure risks. Specifically, this can be done through the following:

*Reusable Non-PFAS Medical Devices:* Adopt catheters, IV lines, and suction tubing made from thermoplastics or elastomers that do not contain PFASs, which are designed for multiple sterilization cycles. This not only limits PFAS waste but also maintains device performance across patient uses.

*Closed-Loop Material Recycling:* Implement hospital-level recycling programs for sterilizable non-PFAS medical plastics. After end-of-life use, materials should be reprocessed and reused within controlled clinical pathways, reducing reliance on virgin fluorinated plastics.

*Informed Procurement Policies:* Integrate circularity criteria into purchasing agreements by favoring products with documented reuse capabilities and PFAS-free certification. Suppliers should provide sterilization protocols that confirm device integrity and safety over repeated use.

These strategies align with sustainable healthcare practices by reducing hazardous waste, lowering PFAS environmental release, and supporting the long-term stewardship of medical materials in neonatal and pediatric settings.

## 8. Conclusions

Per- and polyfluoroalkyl substances (PFASs) are pervasive environmental contaminants with substantial health implications, particularly for pediatric populations. Their chemical persistence, bioaccumulation, and links to a wide array of adverse health outcomes underscore the need for coordinated efforts in exposure monitoring, risk mitigation, and policy development.

In healthcare environments—especially neonatal and pediatric intensive care—PFASs exposure may arise through the prolonged use of medical devices containing fluorinated polymers. Identifying and quantifying PFASs in such devices, developing performance-equivalent PFAS-free alternatives, and promoting transparency in procurement are key to safeguarding vulnerable patients.

In parallel, environmental PFAS contamination continues to disproportionately affect children due to their unique physiological susceptibilities and behavioral exposure patterns. Addressing this requires systemic environmental surveillance, public health education, and pediatric-focused clinical screening programs.

While regulatory agencies have made significant progress in addressing PFASs in drinking water and industrial settings, critical gaps remain concerning medical device-specific regulation. Cross-sector collaboration among researchers, clinicians, manufacturers, and policymakers is essential to close these gaps and facilitate safer clinical practices.

Finally, integrating principles of circular economy—such as reusable PFAS-free medical devices, closed-loop recycling systems, and sustainable procurement frameworks—can significantly reduce PFAS waste and exposure in clinical settings. This approach complements broader environmental and public health goals and supports a resilient, sustainable healthcare system.

## Figures and Tables

**Table 1:** Common PFASs used in IVD platforms.

Fluoropolymers	Fluoroelastomers	Fluorinated Surfactants/Solvents
Polytetrafluoroethylene (PTFE)	Fluoroelastomers (FKM/FPM)	Hexafluoro-2-propanol (HFP)
Fluorinated ethylene propylene (FEP)	Perfluoroelastomers (FFKM/FFPM)	Trifluoroacetic acid (TFA)

**Table 2:** Common PFASs used in medical devices and their applications.

PFAS Category	Compound/Class	Common Applications
**Fluorinated Polymers and Copolymers**	Polytetrafluoroethylene (PTFE)	Catheters, sutures, vascular grafts, and implant coatings
	Fluorinated ethylene propylene (FEP)	Tubing, filter membranes, and drug delivery systems
	Polyvinylidene fluoride (PVDF)	Tubing, membranes, and infusion systems
	PVDF-Hexafluoropropylene) (PVDF-HFP)	Multilayer films, drug packaging, and infusion bags
	Perfluoropolyethers (PFPEs)	Lubricated device components (e.g., moving joints, seals)
	Perfluorinated acrylates (C6–C14)	Hydrophobic coatings and anti-fouling surface treatments
**Surface Modifications**	Proprietary fluoropolymer coatings	Biocompatibility enhancement and reduced microbial adhesion on medical device surfaces

**Table 3:** Specialty fluorinated lubricants and suture materials used in medical devices.

PFAS Type	Compound/Class	Medical Applications
**Fluoroelastomers**	FKM/FPM fluoroelastomers	Seals, O-rings, and flexible components in surgical and diagnostic instruments
	FFKM/FFPM perfluoroelastomers	High-performance sealing systems in sterilizable and chemically resistant environments
**Suture and Graft Materials**	PTFE-based sutures and vascular grafts	Soft tissue suturing, vascular repair, low reactivity, and improved knot security
	PVDF-based sutures	Durable suturing, enhanced mechanical strength and biocompatibility
**Semi-fluorinated Alkanes**	1-(Perfluorohexyl)octane; 1-(Perfluorobutyl)pentane	Ophthalmic surgery (e.g., retinal procedures) and biocompatible surgical lubricants

**Table 4:** Relative PFAS exposure routes in pediatric patients.

Exposure Route	Pathway	Magnitude of Exposure *	Confidence Level *	Notes
**Environmental–Drinking water**	Ingestion of contaminated tap or bottled water	High	High	Children consume more water per kg than adults, increasing exposure risk [53,54]
**Environmental–Dust/Soil**	Ingestion during hand-to-mouth activity	Moderate	High	Dust exposures are significant due to behavioral factors [47]
**Breastfeeding**	Transfer of PFASs through human milk	High (infancy)	High	Exclusive breastfeeding can increase serum PFAS by ~30%/month [53,55]
**Prenatal (gestational transfer)**	Maternal–fetal transfer via placenta	Moderate	Moderate	Contributes significantly to exposure at birth [53,56,57]
**Medical devices**	PFAS-containing bottles, tubing, catheters, and ECMO lines	Low–Moderate	Low	Not well quantified, but clinically relevant [22,27,58,59]

* The categorization of PFAS exposure routes by magnitude of exposure and confidence level was based on a synthesis of available biomonitoring data, mechanistic studies, and clinical observations from recent peer-reviewed literature. “High magnitude” designations (e.g., drinking water, breastfeeding) reflect consistent findings of elevated serum PFAS levels in exposed children, especially in infancy, with strong temporal or dose–response correlations [18,47,53,54,55,56]. “Moderate” levels were assigned to prenatal (gestational) transfer and dust ingestion, supported by cohort data showing significant but variable PFAS levels in cord blood or from behavioral ingestion [47,54]. “Confidence level” reflects the robustness and reproducibility of available data. High confidence was assigned when multiple studies with consistent outcomes were available, while low confidence was reserved for routes like medical devices, where clinical relevance is suspected but data remain scarce or indirect [22,27,57,58,59].

## Data Availability

Not applicable.

## Abbreviations

The following abbreviations are used in this manuscript:

PFASs	Per- and polyfluoroalkyl substances
ICU	Intensive care units
PTFE	Polytetrafluoroethylene
PVDF	Polyvinylidene fluoride
IVD	In vitro diagnostic
HFP	Hexafluoro-2-propanol
TFA	Trifluoroacetic acid
FEP	Fluorinated ethylene propylene
PCTFE	Poly(chlorotrifluoroethylene)
ETFE	Ethylene tetrafluoroethylene
FKM/FPM	Fluoroelastomers
FFKM/FFPM	Perfluoroelastomers
PVDF	Polyvinylidene fluoride
PVDF-HFP	Poly(vinylidene fluoride-hexafluoropropylene)
C6–C14	Perfluorinated acrylates
AFFFs	Aqueous film-forming foams
MAFLD	Metabolic-associated fatty liver disease
NAFLD	Non-alcoholic fatty liver disease
EU	European Union
ECHA	European Chemicals Agency
REACH	Registration, Evaluation, Authorisation, and Restriction of Chemicals
EPA	Environmental Protection Agency
CERCLA	Comprehensive Environmental Response, Compensation, and Liability Act
MCLs	Maximum contaminant levels
WHO	World Health Organization
PPAR	Peroxisome proliferator-activated receptor
PFOS	Perfluorooctane sulfonate
PFOA	Perfluoroalkyl and polyfluoroalkyl substances
TSCA	Toxic Substances Control Act
MDR	Medical device regulation
MDD	Medical device directive
NIS	Na+/I- symporter
EtFOSAA	N-ethyl perfluorooctane sulfonamido acetic acid
PPAR-α	Peroxisome proliferator-activated receptor alpha
ASD	Autism spectrum disorder
ADHD	Attention-deficit/hyperactivity disorder
SHBG	Sex hormone binding globulin
OWO	Obesity and overweight
PBPK	Physiologically based pharmacokinetic
MAFLD	Metabolic-associated fatty liver disease
NAFLD	Non-alcoholic fatty liver disease
AAC	Aortic calcification
AFFF	Aqueous film-forming foams
MeSH	Medical subject headings
